# On the prevalence of constipation and fecal incontinence, and their co-occurrence, in the Netherlands

**DOI:** 10.1007/s00384-016-2722-3

**Published:** 2016-12-02

**Authors:** Rob J. Meinds, Maxime M. van Meegdenburg, Monika Trzpis, Paul M.A. Broens

**Affiliations:** 1 Department of Surgery, Division of Pediatric Surgery, University of Groningen, University Medical Center Groningen, Hanzeplein 1, PO Box 30 001, 9700 RB Groningen, the Netherlands; 2 Department of Surgery, Anorectal Physiology Laboratory, University of Groningen, University Medical Center Groningen, Groningen, the Netherlands

**Keywords:** Constipation, Fecal incontinence, Prevalence, Population

## Abstract

**Purpose:**

Numerous studies have investigated the prevalence of constipation and fecal incontinence (FI) in the general population and, even though these disorders are known to co-occur, they were studied independently of each other. Our aim was to investigate the prevalence of constipation and FI, and their co-occurrence, in the general population in the Netherlands.

**Methods:**

We studied a cross-section of the Dutch population (*N* = 1259). All respondents completed the Groningen Defecation & Fecal Continence checklist. We defined constipation and FI in accordance with the Rome III criteria.

**Results:**

We found that 24.5% (95% CI, 22.1–26.8) suffered from constipation, 7.9% (95% CI, 6.4–9.4) suffered from FI, and 3.5% (95% CI, 2.5–4.5) suffered from both disorders. Constipated respondents were 2.7 times more likely to suffer from FI than non-constipated respondents (95% CI, 1.8–4.0). Moreover, 48.7% of the respondents with constipation, 35.0% with FI, and 38.6% in whom the disorders co-occurred qualified their bowel habits as either “good” or “very good”. We found that 49.4% of the respondents with constipation and 48.0% with FI had not discussed their complaints with anyone.

**Conclusions:**

Constipation and FI, isolated or co-occurring, are common disorders in the general population, even in young and healthy respondents. Since constipation and FI often co-occur, we recommend that patients who seek medical attention for either disorder should be examined for both. Moreover, constipation and/or FI are not always identified appropriately by patients. Therefore, physicians should take the initiative to diagnose and treat these disorders.

**Electronic supplementary material:**

The online version of this article (doi:10.1007/s00384-016-2722-3) contains supplementary material, which is available to authorized users.

## Introduction

Bowel habits and associated disorders, such as constipation and fecal incontinence (FI) are very private and are therefore rarely discussed by patients. Even in the consulting room these matters are seldom addressed if they are not the primary reason for visiting a doctor. This could be due to embarrassment on the part of the patient or due to lack of knowledge and/or interest on the part of the physician [1]. Alternatively, patients could simply be unaware of the fact that their defecation habits might not be considered normal.

Due to both ignorance and the stigma attached to this subject, the impact of constipation and FI on the general population has long been unclear. Over the last decades, however, numerous studies were performed investigating the bowel habits in the general population in various countries including Taiwan, USA, Iran, Greece, Italy, and Australia. These studies found that constipation and FI have a prevalence of 2.5 to 22.8% [2–9] and 7.2 to 12.1% [8–11], respectively. Albeit, these studies, considered constipation and FI independently of each other and neglected to investigate the co-occurrence of the two disorders. While the effect of constipation on FI is well-known in children and geriatric patients [12], to date, the frequency of the co-occurrence of constipation and FI has not been investigated in the general population. Nor has a population-based study on bowel habits ever been performed in the Netherlands, even though such a study is vital for assessing the impact and burden of these disorders on Dutch society.

Our primary aim, therefore, was to investigate the prevalence of constipation and FI in the Dutch population, as well as the co-occurrence of the two disorders. We hypothesize that, given the high prevalence of both disorders, a significant group of the population will suffer from both disorders. Our second aim was to investigate how respondents qualified their own health regarding the ability to hold and pass stools, and how often they sought medical help for their defecation complaints.

## Methods

### Study design

We examined a cross-section of the Dutch population between September 1 and November 1, 2015. In order to obtain representative data, we commissioned Survey Sampling International (Rotterdam, the Netherlands), a company specialized in performing surveys, to draw a population-based sample from a database of respondents. The participants in this database were sent a link that enabled them to fill out the Groningen Defecation and Fecal Continence (DeFeC) checklist on their computer (supplementary data). The lower age that limits for inclusion was set at 18 years, while there was no upper age limit. Out of a total of 3031 eligible respondents who started filling out the checklist, 1642 (54.2%) filled it out completely. Subsequently, a random selection of these checklists was made by Survey Sampling International to arrive at a representative cohort, equally distributed regarding gender, region, and age according to the population pyramid of the Netherlands as reported by Statistics Netherlands [13]. By doing so, 1259 out of 1642 (76.7%) checklists were included in our analysis.

### Assessment of constipation and fecal incontinence

We defined constipation according to the Rome III criteria for constipation [14]. These criteria consist of the following items: straining, lumpy or hard stools, incomplete evacuation, anorectal blockage, manual maneuvers to facilitate defecation, and reduced stool frequency. In order to meet the criteria for constipation, the respondents had to have at least two of the aforementioned complaints, plus rarely having loose stools without the use of laxatives. We also defined FI according to the Rome III criteria for FI, i.e., recurrent uncontrolled passage of fecal material (including soiling) at least several times per month, for the last 3 months [15]. We performed a subanalysis to determine from which type of FI the respondents suffered. Soiling was defined as the loss of small amounts of feces or staining of underwear, urge FI as being unable to reach the toilet in time after feeling an urge sensation, liquid stool FI as loss of watery stools or diarrhea, and solid stool FI as loss of large amounts of solid feces without having felt urge.

### Assessment of bowel-related quality of life and help-seeking behavior

We also asked respondents how they would qualify their ability to hold and pass stools. Furthermore, if they suffered from constipation, FI or both, we asked whether the respondents ever talked about their defecation problems to someone (e.g., family, friends, general practitioner, medical specialist, or other).

### Data analysis

In order to analyze the prevalence of constipation and FI at different ages, we divided the respondents into five groups on the basis of their age percentiles: 18 to 34, 35 to 46, 47 to 55, 56 to 64, and 65 to 85-year-olds. We first analyzed the entire group of respondents, irrespective of whether they suffered from any comorbidity known to influence defecation pattern and fecal continence. By so doing, we defined the true rate of constipation and FI of the total Dutch population. Subsequently, we performed a subanalysis to define the rate of constipation and FI in the “healthy” Dutch population, i.e., that part of the population which did not experience any disease that could negatively influence bowel habits and continence. Thus, we excluded respondents who had a history of bowel surgery (e.g., intestinal resection, perianal fistula operation, anal sphincter operation, hemorrhoid operation, prostate operation) or respondents who suffered from somatic diseases that could influence their bowels, such as rectal prolapse, inflammatory bowel diseases, diabetes, cerebral stroke, neurological disorders (e.g., spinal cord injury, multiple sclerosis), slow transit constipation, or congenital disorders (e.g., anorectal malformation, Hirschsprung’s disease, sacrococcygeal teratoma, or spina bifida).

### Statistical analysis

Data were analyzed with SPSS 23.0 for Windows (IBM SPSS Statistics, IBM Corporation, Armonk, NY). Proportions were reported as prevalence percentages with the corresponding 95% confidence intervals (CIs). Comparison between proportions was made using Pearson’s chi-square test. Proportions were additionally used to calculate odds ratios (ORs) between groups, reported with the corresponding 95% CIs. The probabilities of constipation and/or FI were defined by the number of respondents with constipation and/or FI, respectively, divided by the total number of respondents at any age. The relationship between age and the probability of constipation and FI was evaluated by spline regression analysis using Stata 14 (StataCorp, College Station, TX). Two-sided *p* values of less than 0.05 were considered statistically significant. Figures were generated using GraphPad Prism 5.04 (GraphPad Software Inc., La Jolla, CA).

## Results

### Respondent characteristics

A total of 1259 checklists were completed entirely by 46.0% (*n* = 579) male respondents and 54.0% (*n* = 680) female respondents, with a median age of 49 years (Table 1). At the time of filling out the checklist, 50.4% (*n* = 635) of the respondents were either unemployed or did not hold a job for various reasons, such as household commitments/raising children (5.9%), pre-pension/pension (20.3%), study (4.0%), health-related problems (9.9%), or involuntary unemployment (10.5%). A total of 19.9% (*n* = 251) of the respondents reported suffering from somatic diseases that could potentially influence their bowel patterns and fecal continence or reported having a history of bowel surgery.

**Table 1:** Respondent characteristics

	Number	Percent
Overall	1259	100.0
Gender		
Male	579	46.0
Female	680	54.0
Age (years)		
18–34	273	21.7
35–46	256	20.3
47–55	253	20.1
56–64	234	18.6
65–85	243	19.3
Highest educational level		
Primary	260	20.7
Secondary	505	40.1
Tertiary	494	39.2
Employed		
Yes	624	49.6
No	635	50.4
Residence		
Rural	436	34.6
Urban	823	65.4
Co-morbidities influencing bowel pattern		
Yes	251	19.9
No	1008	80.1

### Prevalence and probability of constipation

**Fig. 1:**
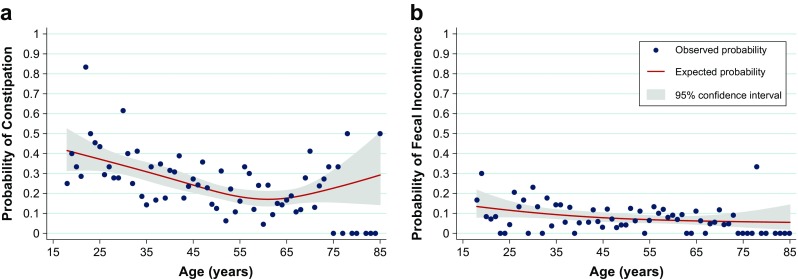
The probability of constipation and fecal incontinence plotted against the age of the respondents. **a** The probability of constipation gradually decreased to a minimum value of approximately 0.17 at 61 years, after which the probability increased as respondents’ ages increased. **b** The overall probability of FI did not change with increasing respondents’ age

Firstly, we analyzed the prevalence of constipation for gender and different age groups (Table 2). Overall, 24.5% (95% CI, 22.1–26.8) of the respondents suffered from constipation. Females were 1.8 times more likely to suffer from constipation than males (95% CI, 1.4–2.3). Moreover, the prevalence of constipation decreased with increasing age (Table 2, *p* < 0.001). Because we found a significant difference in the prevalence of constipation between different age groups, we analyzed how the probability of constipation changed with age (Fig. 1a). The probability of constipation gradually decreased to a minimum of approximately 0.17 at 61 years. After this initial decrease we found an increase in the probability of constipation as respondents’ ages increased beyond 61 years of age.

**Table 2:** Prevalences of constipation and fecal incontinence in the Dutch population

		Constipation			Fecal incontinence			Constipation and fecal incontinence		
	Total *n*	%	95% CI	*p* value	%	95% CI	*p* value	%	95% CI	*p* value
Overall	1259	24.5	22.1–26.8		7.9	6.4–9.4		3.5	2.5–4.5	
Gender				<0.001			1.00			0.81
Male	579	18.8	15.6–22.0		7.9	5.7–10.2		3.6	2.1–5.2	
Female	680	29.3	25.8–32.7		7.9	5.9–10.0		3.4	2.0–4.7	
Age (years)				<0.001			0.11			0.004
18–34	273	36.3	30.5–42.0		11.0	7.3–14.7		7.0	3.9–10.0	
35–46	256	26.6	21.1–32.0		9.0	5.5–12.5		3.9	1.5–6.3	
47–55	253	19.0	14.1–23.8		5.5	2.7–8.4		2.0	0.2–3.7	
56–64	234	19.2	14.1–24.3		8.1	4.6–11.6		1.3	−0.2–2.7	
65–85	243	19.8	14.7–24.8		5.8	2.8–8.7		2.9	0.8–5.0

### Prevalence and probability of fecal incontinence

Secondly, we analyzed the prevalence of FI in different age groups (Table 2). Overall, 7.9% (95% CI, 6.4–9.4) of the respondents suffered from FI. There was no statistically significant difference in the prevalence of FI between males and females (*p* = 0.998), nor between the age groups (*p* = 0.114*)*. We did, however, notice a slight decrease in the prevalence of FI with increasing age in the younger age groups (Table 2). Thus, we also analyzed whether the probability of FI changed with age (Fig. 1b). We found that the overall probability of FI did not change significantly with increasing age.

### Co-occurrence of constipation and fecal incontinence

Lastly, we analyzed the prevalence of the co-occurrence of constipation and FI in the different age groups (Table 2). We found that 3.5% (95% CI, 2.5–4.5) of the respondents suffered from both disorders. Moreover, we observed that the two disorders co-occurred significantly more often in the younger age groups, namely 7.0% (95% CI, 3.9–10.0) in the 18 to 34-year-olds versus 2.9% (95% CI, 0.8–5.0) in the 65 to 85-year-olds (*p* = 0.004, Table 2). In addition, we found that constipated respondents were 2.7 times more likely to suffer from FI than non-constipated respondents (95% CI, 1.8–4.0) (Table 3). Nearly three-quarters of the respondents, i.e., 71.1% (*n* = 895), experienced neither constipation nor FI.

**Table 3:** Types of fecal incontinence in respondents with constipation

	Constipation		
	No	Yes	
	*n* (%)	*n* (%)	*p* value
Overall	951 (100.0)	308 (100.0)	
Fecal incontinence	56 (5.9)	44 (14.3)	*<0.001*
Types of fecal incontinence^a^			
Soiling	45 (4.7)	34 (11.0)	*<0.001*
Solid stool	11 (1.2)	25 (8.1)	*<0.001*
Urge	19 (2.0)	22 (7.1)	*<0.001*
Liquid stool	16 (1.7)	22 (7.1)	*<0.001*

^a^Respondents often suffered from multiple types of fecal incontinence

We also analyzed which types of FI (soiling, solid stool, urge, and liquid stool) were suffered by respondents with constipation (Table 3). All types of FI were seen significantly more often in constipated respondents than in respondents who did not suffer from constipation (*p* < 0.001). Subsequently, we analyzed the prevalence of the different constipation complaints incorporated in the Rome III criteria for constipation and compared respondents with and without FI (Table 4). Straining and incomplete evacuation were the constipation complaints most frequently experienced by respondents with FI, namely by 50.0% (50 out of 100) and 54.0% (54 out of 100), respectively. Manual maneuvers and reduced stool frequency were experienced least, namely by 25.0% (25 out of 100) and 25.0% (25 out of 100) respondents, respectively. Nearly all the constipation complaints that were analyzed, except lumpy or hard stools, were seen significantly more often in respondents who suffered from FI than in respondents who did not suffer from FI (*p* < 0.001, Table 4).

**Table 4:** Constipation complaints in respondents with fecal incontinence

	Fecal incontinence		
	No	Yes	
	*n* (%)	*n* (%)	*p* value
Overall	1159 (100.0)	100 (100.0)	
Constipation	264 (22.8)	44 (44.0)	*< 0.001*
Constipation complaints			
Straining	333 (28.7)	50 (50.0)	*< 0.001*
Lumpy or hard stools	436 (37.6)	40 (40.0)	*0.64*
Incomplete evacuation	278 (24.0)	54 (54.0)	*< 0.001*
Anorectal blockage	180 (15.5)	36 (36.0)	*< 0.001*
Manual maneuvers	86 (7.4)	25 (25.0)	*< 0.001*
Fewer than three bowel movements per week	89 (7.7)	25 (25.0)	*< 0.001*
Laxative usage at least multiple times per month	62 (5.3)	23 (23.0)	*< 0.001*

Moreover, we analyzed the prevalence of laxative use in respondents with constipation, FI, or both. We found that the use of laxatives, at least several times per month, was significantly higher in patients in whom constipation and FI co-occurred (43.2%) when compared to those with only constipation (21.4%, *p* = 0.002) or FI (23.0%, *p* = 0.014).

### Defecation frequency and stool consistency

**Fig. 2:**
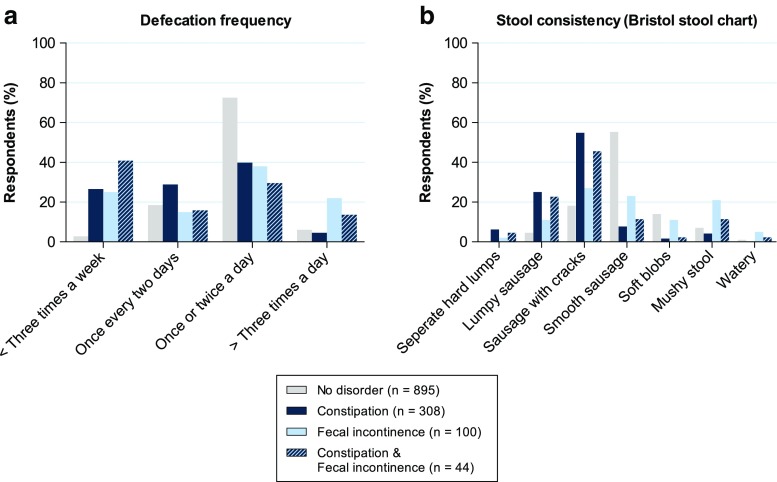
Defecation frequency and stool consistency. **a** The frequency of bowel movements. **b** The consistency of stools (according to the Bristol stool chart)

We also investigated defecation frequency (Fig. 2a) and stool consistency (Fig. 2b) in patients with no defecation disorder, constipation, FI, and in whom constipation and FI co-occurred.

The respondents who suffered from constipation (*n* = 308) had a significantly lower defecation frequency than the respondents with no defecation disorder (*p* < 0.001, Fig. 2a). Only 26.6% of the respondents with constipation had less than three bowel movements per week, while the defecation frequency of the majority (68.8%) was normal, i.e., every other day to twice a day.

Respondents with FI (*n* = 100) were more likely to have either a low frequency or a high frequency of bowel movements (25.0 and 22.0%, respectively) than respondents with no defecation disorder (2.8 and 6.1%, respectively, *p* < 0.001). Even so, the defecation frequency of the majority (53.0%) of the respondents with FI was normal.

Interestingly, of the respondents in whom constipation and FI co-occurred (*n* = 44), a large portion (40.9%) had a low frequency of bowel movements (less than three per week), which was significantly different to the group of respondents with no defecation disorder (2.8%, *p* < 0.001).

We also investigated stool consistency according to the Bristol stool chart (Fig. 2b). Overall, stool consistency of constipated respondents was harder than that of respondents with no defecation disorder (*p* < 0.001). Even so, the majority (62.7%) of respondents who suffered from constipation had a normal stool consistency. Respondents who suffered FI had either very hard or very soft (watery) stools (13.0 and 37.0%, respectively) more often than respondents with no defecation disorder (4.5 and 22.0%, respectively, *p* < 0.001). Nevertheless, stool consistency of the majority (50.0%) of the respondents who suffered FI was normal. Lastly, we found that respondents who suffered from both constipation and FI had a hard stool consistency (27.3%) more often than respondents with no defecation disorder (4.5%, *p* < 0.001).

### Respondents’ qualification of bowel habits and help-seeking behavior

**Fig. 3:**
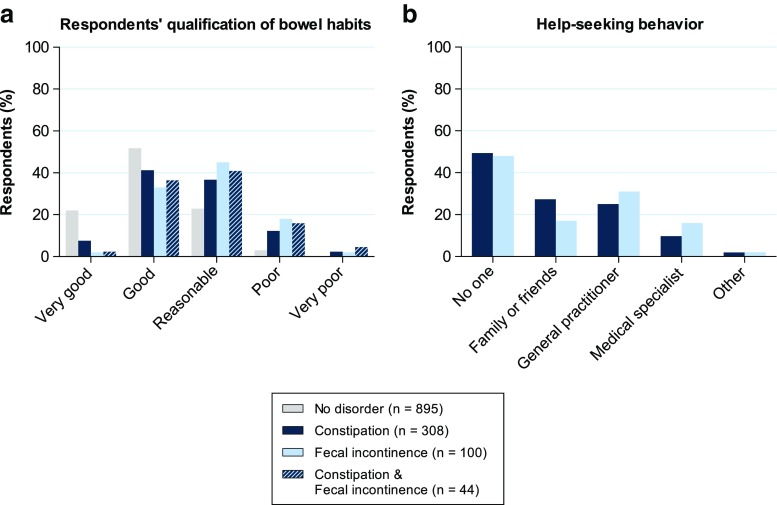
Opinion on bowel habits and help-seeking behavior. **a** Respondents’ qualification of own bowel habits regarding the ability to hold and pass stools. **b** With whom respondents discussed their defecation disorders

At the beginning of the checklist, we asked respondents how they would qualify their ability to hold and pass stools. The answer of 17.6% (*n* = 221) of the respondents was “very good”, 48.3% (*n* = 608) answered with “good”, 27.4% (*n* = 345) with “reasonable”, 6.0% (*n* = 76) with “poor”, and the answer given by 0.7% (*n* = 9) was “very poor”. Additionally, we analyzed these answers for the subgroups of respondents with no defecation disorder, constipation, FI, and for those respondents in whom constipation and FI co-occurred (Fig. 3a). On average, respondents with either constipation, FI, or both, rated their bowel habits significantly lower than the group without a defecation disorder (*p* < 0.001). Nevertheless, 48.7% (150 out of 308) of the respondents with constipation, 35.0% (35 out of 100) of those with FI, and 38.6% (17 out of 44) of those with co-occurring constipation, and FI rated their ability to hold and pass stools as either “good” or “very good”.

Furthermore, we also asked respondents if they ever discussed their constipation or FI problems with anyone, and if so, whom did they speak to (Fig. 3b). Of all constipated respondents, 49.4% (152 out of 308) had never spoken to anyone about their constipation problems. For FI, we found that 48.0% (48 out of 100) had never mentioned their incontinence complaints to anyone.

### Defecation disorders in “healthy” respondents

We also performed an analysis in a “healthy” subgroup (*n* = 1008), i.e., respondents without a history of bowel surgery or somatic disorders that could potentially influence their bowels. The prevalence of constipation in the group of “healthy” respondents was 22.3% (95% CI, 19.7–24.9), and not significantly different from the total group investigated, which was 24.5% (95% CI, 22.1–26.8; *p* = 0.232). By contrast, there was a significant difference in the prevalence of FI between the “healthy” subgroup and the total group, 5.5% (95% CI, 4.1–6.9) versus 7.9% (95% CI, 6.4–9.4; *p* = 0.020), respectively. The co-occurrence of constipation and FI was significantly lower in the “healthy” subgroup (1.9%; 95% CI, 1.0–2.7), than in the total group (3.5%; 95% CI, 2.5–4.5; *p* = 0.020).

We have also separately analyzed respondents with a history of bowel surgery (*n* = 125) and somatic disease (*n* = 173). The prevalence of constipation, FI, and the combination of both disorders was relatively high in respondents with a history of bowel surgery (41.6%, 21.6%, and 14.4%, respectively) and somatic disease (31.8%, 17.9%, and 9.8%, respectively). The difference in the prevalence of the aforementioned disorders between the two groups of respondents was not statistically significant (*p* = 0.082, *p* = 0.428, and *p* = 0.226, respectively).

## Discussion

This nationwide Dutch survey was the first study on the prevalence of constipation, FI, and the co-occurrence of these two disorders using the Groningen DeFeC checklists. We demonstrated that both constipation and FI occurred frequently in the Dutch population, with a prevalence of 24.5 and 7.9%, respectively. More importantly, we showed that in 3.5% of the population the disorders co-occurred, and that constipated individuals were more likely to suffer from FI.

Even though constipation and FI have been studied extensively in the general population, studies on the co-occurrence of the two disorders are limited to pediatric and geriatric populations and to women who visited gynecologic clinics [12, 16]. In the general adult population, studies only pointed out that certain symptoms of constipation, such as incomplete evacuation, are risk factors for FI [12]. Our study, however, demonstrated that 3.5% of the general Dutch population suffered from both constipation and FI. This co-occurrence of disorders is seen particularly in 18 to 34-year-old males and females. The relationship between these two disorders could indicate that constipation is a risk factor for FI and that it might play a role in the pathophysiology of FI. This theory is supported by our finding that constipated respondents suffered from FI more often than non-constipated respondents. Furthermore, this theory could also help explain the relatively high prevalence of FI we found in the younger age groups, who suffered from constipation significantly more often than the older age groups.

Three mechanisms have been described as possible causes for the co-occurrence of constipation and FI [12]. Firstly, it is known that in pediatric and geriatric populations, constipation can lead to overflow FI. Secondly, excessive straining, associated with constipation, can lead to pelvic floor denervation and weakness, which could eventually result in FI. Thirdly, rectal evacuatory disorders, such as dyssynergic defecation and rectocele, can lead to incomplete rectal evacuation, resulting in post-defecation leakage. These mechanisms are supported by our results, as we found that respondents with FI suffered from straining and incomplete evacuation complaints significantly more often than respondents without FI. Nevertheless, it is important to perform follow-up studies to elucidate the mechanisms underlying the co-occurrence of constipation and FI in the general population.

Interestingly, when we investigated the defecation frequency and stool consistency of respondents with different defecation disorders, we found that the majority of respondents with a bowel disorder had a normal defecation frequency and stool consistency. This indicates that frequency and consistency are poor predictors of the presence of constipation or FI and are of little value without the addition of more in-depth questions on defecation habits. Additionally, we found that a large portion of respondents who suffered from both constipation and FI had low defecation frequencies (less than three times per week), while this was not the case in respondents who only suffered from constipation. Moreover, respondents in whom constipation and FI co-occurred used significantly more laxatives than those who suffered from either constipation or FI. Since the group of respondents in whom the two disorders co-occurred also suffered significantly more often from other constipation-associated complaints, we hypothesize that this group suffered from a more severe form of constipation.

When asked to comment on their bowel habits, respondents with constipation, FI, or co-occurrence of both disorders qualified their ability to hold and pass stools significantly lower than respondents without a defecation disorder. Nevertheless, 48.7% of the respondents with constipation, 35.0% of those with FI, and 38.6% of those in whom the two disorders co-occurred qualified their ability to hold and pass stools as either “good” or “very good”. Possibly, a considerable part of the population is unaware as to what is considered normal, or abnormal, regarding their own bowel habits or they have become used to the abnormal bowel condition and do, therefore, not recognize it as being a problem. Another interesting finding for patients who suffered either constipation or FI was that 49.4% and 48.0%, respectively, never discussed their defecation problems with anyone. Reasons for these high percentages could be unawareness of the problem and possible treatment options, embarrassment, or even ignorance [17–20]. Since there are good treatment possibilities for constipation and FI, it would seem justified for general practitioners to pay more attention to defecation disorders, even if this is not the primary reason for being consulted.

We found a prevalence of 24.5% for constipation. This is relatively high in comparison to previous Rome II or Rome III criteria-based studies that reported prevalences varying between 2.5% and 22.8% [2–9]. These discrepancies might result from different demographic and geographical features of the populations investigated, such as age and gender distribution and a variation in diet. Moreover, although it was previously reported that FI increases with age, our study did not confirm this findings, for which we offer two explanations. Firstly, we used a digital survey system and, therefore, we possibly included a selection of relatively healthy elderly respondents. Secondly, and more importantly, the relatively high prevalence of FI in the younger age groups could have resulted from the significantly higher prevalence of the co-occurrence of constipation and FI in these groups in comparison to the older age groups. Based on our daily clinical experience, we expect that the severity of fecal incontinence might increase when people get older. This issue however, requires further investigation, where the correlation between age and severity of fecal incontinence should be analyzed.

To investigate whether defecation disorders were predominantly caused by comorbidities, we performed a subanalysis. On the one hand, we found that the prevalence of FI was significantly lower in the “healthy” subgroup, i.e., respondents without a history of bowel surgery or somatic disorders, than in the total study group. On the other hand, we found no significant difference regarding the prevalence of constipation when comparing the “healthy” subgroup to the total population. Thus, it would seem that somatic disorders and bowel surgery might constitute considerably larger risk factors for developing FI than for developing constipation.

The main limitation of this study was the possible selection bias towards healthy elderly respondents by performing a digital survey. It is most likely, therefore, that as far as the elderly are concerned, our study underestimated the prevalence of constipation, and more importantly, the prevalence of FI. On the other hand, by performing this survey digitally, we were able to include a large and representative group of the Dutch population reflecting the gender and age proportions as found in the overall population. Furthermore, a response rate of 54% may be considered low. This might be explained by the subject and length of the checklist. This low response rate could, however, also have biased the results.

## Conclusions

In accordance with our hypothesis, we found that a relatively large part of the Dutch population suffered from both constipation and FI. Moreover, even young and healthy respondents often suffered from defecation disorders. The increased likelihood of FI in constipated respondents leads us to conclude that constipation could be considered a causative factor of FI. We, therefore, recommend that patients seeking medical attention for either constipation or FI should be examined for both disorders, since they often co-occur. Remarkably, a large part of the population was unaware of the fact that their bowel habits could not to be considered normal, and a significant number of these respondents had never sought medical attention. These findings warrant further investigations in order to improve patient awareness and healthcare for constipation and FI.

## Electronic supplementary material

ESM 1 (PDF 386 kb)
